# Impact of COVID-19 Pandemic on In-Hospital Mortality in Patients Without SARS-CoV-2 Infection in an Internal Medicine Ward of a Tertiary Care Hospital in Portugal

**DOI:** 10.7759/cureus.32059

**Published:** 2022-11-30

**Authors:** Ana Rita Ramalho, Ana Cristina Mendes, Guilherme Camões, Ricardo Roque, Pedro Moura, António Mateus-Pinheiro, Adriana Dias, Andreia Fernandes, Joana Guimarães, João Faria, José Magalhães, José Pedro Fernandes, Pedro Fragoso, João Porto, José Moura, Armando Carvalho, Lèlita Santos

**Affiliations:** 1 Internal Medicine, Coimbra Hospital and University Center, Coimbra, PRT; 2 Oncology, Portuguese Institute of Oncology of Coimbra, Coimbra, PRT; 3 Hematology, Coimbra Hospital and University Center, Coimbra, PRT; 4 Pulmonology, Coimbra Hospital and University Center, Coimbra, PRT; 5 Endocrinology, Portuguese Institute of Oncology of Coimbra, Coimbra, PRT; 6 Cardiology, Coimbra Hospital and University Center, Coimbra, PRT; 7 Physical Medicine and Rehabilitation, Coimbra Hospital and University Center, Coimbra, PRT; 8 Nephrology, Coimbra Hospital and University Center, Coimbra, PRT

**Keywords:** covid-19 retro, internal medicine, prediction model, hospital mortality, sars-cov-2, covid-19

## Abstract

Introduction: Despite the emergence of a new worldwide cause of death related to COVID-19, several studies have hypothesized that the international mortality rate attributed to non-COVID-19 causes was significantly higher during the COVID pandemic, questioning whether this excess in mortality is related only to COVID-19 or to the difficulties that the healthcare systems faced during the pandemic. Therefore, understanding the impact of the COVID-19 pandemic on the prognosis of patients without severe acute respiratory syndrome coronavirus 2 (SARS-CoV-2) infection is a major unmet need as this was overshadowed by the overwhelming number of patients with SARS-CoV-2.

Methods: This is a retrospective, cross-sectional, observational study in the internal medicine non-COVID-19 wards of a tertiary care hospital in Portugal. A total of 2021 patients without SARS-CoV-2 infection admitted between March and May of 2019 and 2020 were included. For each patient, we collected information regarding demographic characteristics, emergency department admission information, hospitalization information, date of discharge or death, health comorbidities, and current medication.

Results: Data from 1013 patients in 2019 and 1008 patients in 2020 was analyzed. The patients' demographic characteristics, health comorbidities, and current medications were distributed in similar patterns in the two studied periods. There was a statistically significant difference in the in-hospital mortality in patients without SARS-CoV-2 infection between 2019 and 2020 (12% vs 17%, p-value < 0.001) and in admission severity in hospitalized patients without SARS-CoV-2 infection between 2019 and 2020 (0.9 vs 0.6, p-value < 0.001).

Conclusion: Our work showed a statistically significant increase in in-hospital mortality during the COVID-19 pandemic in patients without SARS-CoV-2 infection, which was not apparently explained by differences in the characteristics of hospitalized patients. As this is one of the first works describing the silent impact of the COVID-19 pandemic in Portugal, we believe it holds an important value in the provision of bases for building up future health policies in case of new COVID-19 outbreaks or other medical emergencies.

## Introduction

The year 2020 will be remembered as the first year of the COVID-19 pandemic, which affected both developed and developing countries, impacting both healthy people and those with pre-existing illnesses. It caused a significant burden and was a challenge to the healthcare systems worldwide. With approximately 390,000,000 cases and 5,700,000 deaths attributed to COVID-19 on an international level, the COVID-19 pandemic is a current priority of governments worldwide [1].

Notwithstanding the emergence of a new cause of death, several national and international studies have hypothesized that the mortality rate was significantly higher during this period as compared to the previous years, raising the question of whether this excess was caused directly by COVID-19 or by the difficulties that healthcare systems faced during this pandemic [2-5].

In fact, little is known about the silent victims of the COVID-19 pandemic. Since the outbreak, patients lost follow-up for their chronic diseases due to rescheduling of their doctor appointments, unavailability of transport, or fear of leaving their homes and being infected by the virus [5]. There is strong evidence that the delay in hospitalization due to the fear of COVID-19 is presumed to have a negative impact on many clinical conditions requiring timely treatment [6]. In addition to doctor’s appointments being canceled, elective surgeries and pharmacist’s appointments were postponed. This caused delays in receiving medications for their underlying medical problems. Furthermore, healthcare services prioritized COVID-19 patients, and healthcare professionals were recruited from their former clinical assistance, leaving fewer professionals available for the provision of care for non-COVID-19 patients [5,7]. According to some studies, diabetes, chronic obstructive pulmonary disease, and hypertension are among the diseases that suffered a higher impact in the provision of care due to the pandemic [5,8]. Therefore, understanding the impact of the COVID-19 pandemic in patients without severe acute respiratory syndrome coronavirus 2 (SARS-CoV-2) infection is a major unmet need.

There are published studies that report a significant decrease in the number of patients admitted to the emergency department for non-serious conditions and low-priority codes as well as an increase in hospitalization rates, which suggests a greater severity of patients referred to the emergency department [9,10]. Portuguese studies also noted a decrease in the number of admissions to the emergency department for non-respiratory diseases, which is in accordance with international works [7,9-11]. Additionally, preliminary results from the National School of Public Health showed an increase of 1.255 deaths in Portugal between March 16, 2020, and April 14, 2020. When compared to the average mortality observed in the last 10 years, the authors hypothesized that only 49% of the excess mortality was directly attributed to SARS-CoV-2 infection [2]. What has not been studied yet, and what we propose to do, is to assess whether and to what extent the number of deaths not registered as COVID-19-related has increased compared to what was observed in the absence of the virus. We aim to do so by investigating what organizational changes were applied and how they may have impacted the prognosis of non-COVID-19 patients. We also intend to compare the differences in health comorbidities and chronic medication before and during the pandemic. If any difference is present, then we would determine if there was a correlation with outcomes in both time periods and compare predictive models of in-hospital mortality between the years in analysis.

This work represents an effort to study in-hospital mortality of patients without SARS-CoV-2 infection. We hypothesize that in patients without SARS-CoV-2 infection, there was a significant increase in in-hospital mortality and that this was not explained by the differences in patients’ characteristics. We hope that our work is helpful for further understanding the real impact of the COVID-19 pandemic on the Portuguese Health System, particularly in non-intensive care settings and among the elderly.

## Materials and methods

Study design

Our study is a retrospective, cross-sectional, observational study conducted in the internal medicine non-COVID-19 wards of the Coimbra Hospital and University Center, EPE, Coimbra, Portugal, one of the main tertiary care hospitals in Portugal. Our data was collected from Portuguese health information systems.

The project was approved by the Ethics Committee of Coimbra Hospital and University Center, EPE, Coimbra, Portugal. The confidentiality of the data was respected by utilizing the anonymity of the data in the database, in accordance with the General Data Protection Regulation (GDPR) of the European Union and Law No. 58/2019 of the Diary of the Republic of Portugal.

Sample

Patients without SARS-CoV-2 infection admitted to the referred hospital's internal medicine ward between March and May 2020 (our study group) and the same period in 2019 (our control group) were included. Between March and May 2020, all patients were tested for SARS-CoV-2 infection before hospitalization. The inclusion and exclusion criteria mentioned in Table 1 were considered.

**Table 1:** Inclusion and exclusion criteria SARS-CoV-2: Severe acute respiratory syndrome coronavirus 2. ^1^ Patients were kept in the "buffer ward" while awaiting the results of their SARS-CoV-2 infection test, which at the time took 48 hours to become available.

Inclusion Criteria	Exclusion Criteria
Patients without SARS-CoV-2 infection admitted to the internal medicine wards between March and May 2020.	Patients with documentation of SARS-CoV-2 infection during hospitalization.
Patients admitted to the internal medicine wards between March and May 2019.	Patients with elective hospitalization that lasted less than 24 hours.
	Patients without clinical records.
	Patients who died in the buffer ward^1^ before being transferred to an Internal Medicine ward.

The primary endpoint measured was in-hospital mortality in patients without SARS-CoV-2 infection.

Data collected and outcomes

The data collected included the subject’s demographic characteristics (age and gender), emergency department information (vital signs and severity at admission trough calculation of quick sequential organ failure assessment [qSOFA] - a score used to identify patients that are at a greater risk for a poor outcome), hospitalization information (vital signs, functional status at hospital admission using the Katz index of independence in activities of daily living, length of hospital stay, day of hospital admission [categorizing them as admission at week days or weekends], time of hospital admission [categorizing them as admission between 12 pm and 8 am, between 8 am and 4 pm, and between 4 pm and 12 pm], need for differentiated care in an intensive care unit, and stay in a buffer ward), date of discharge or death, health comorbidities (presence of heart failure, diabetes mellitus, chronic pulmonary disease/asthma, hypertension, chronic kidney disease, dementia, cerebrovascular disease, and cancer), and home medication (diuretics, beta-blockers, calcium channel blockers, statins, antiplatelets, anticoagulants, inhibitors of the renin-angiotensin system, oral hypoglycemic medications, insulin, and proton pump inhibitors).

Statistical analysis

Descriptive statistics, such as absolute and relative frequencies, mean, standard deviations, and medians, were used to summarize univariate variables. Univariate and bivariate analyses using the Chi-square test or Fisher's exact test were performed. Two sample t-test and z-test were used to assess the differences between the two groups. Correspondent tests were used for non-parametric variables.

To understand the independent predictors of in-hospital mortality, multivariable logistic regression was used. Receiver operating characteristic (ROC) curve analysis was used to assess the performance of the final model. A p-value of 0.05 or less was considered statistically significant. Statistical analysis was performed using STATA v16 (StataCorp LLC, College Station, TX).

## Results

Characteristics of included patients

**Figure 1:**
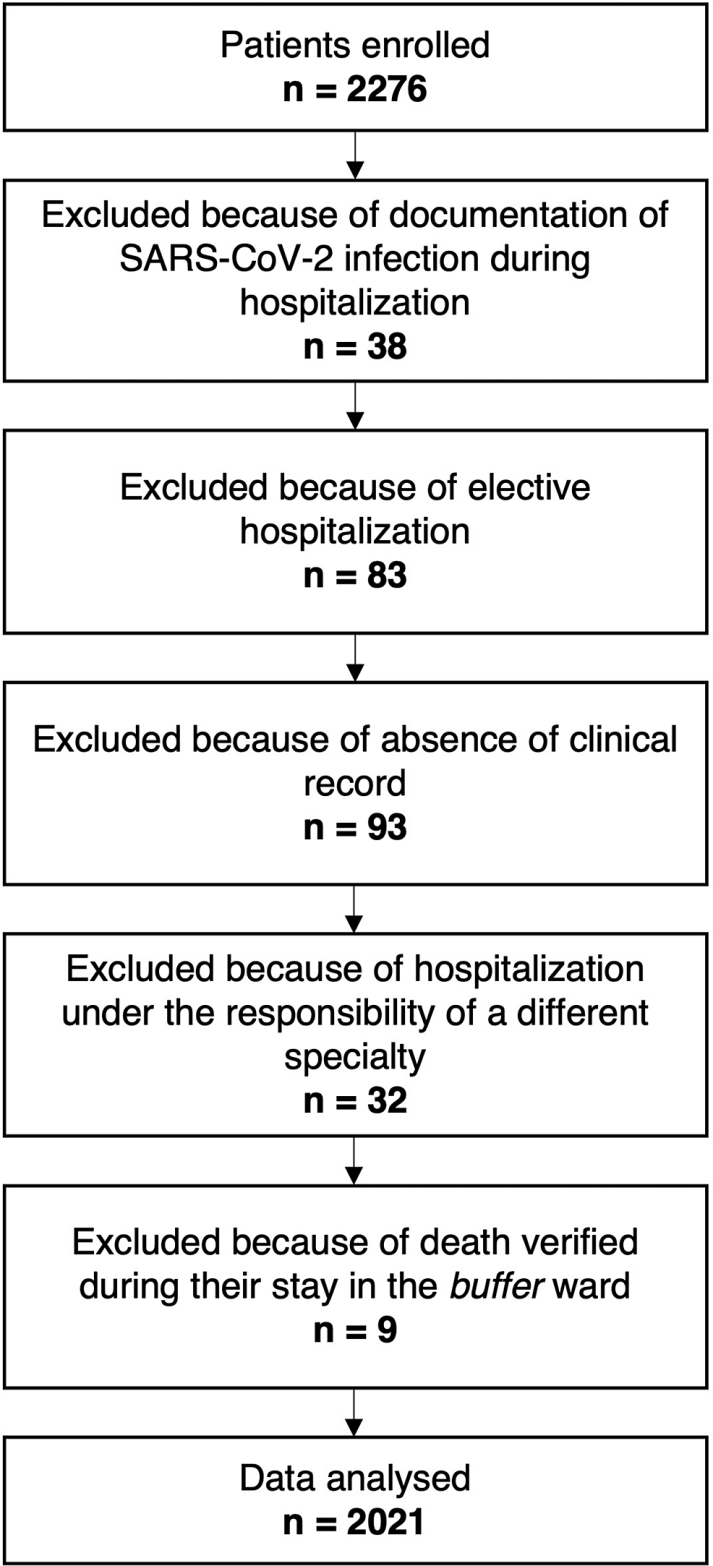
Flowchart of the patients throughout the study SARS-CoV-2: Severe acute respiratory syndrome coronavirus 2.

Of the 2276 patients initially enrolled, 2021 were included in the study (Figure 1).

Of the subjects studied in 2019 and 2020, 508 (50%) and 554 (55%), respectively, were female. The average age (standard error) of the patients was 78 years (0.43) in 2019 and 79 years (0.45) in 2020. Regarding the patients’ functional status at hospital admission, 438 (43%) had a Katz index under 3 and 334 (33%) were fully autonomous in 2019; and 357 (36%) had a Katz index under 3 and 310 (31%) were fully autonomous in 2020. Considering the severity of clinical presentation at hospital admission, 402 (40%) had a quick sequential organ failure assessment(qSOFA) score of 0 in 2019, and 558 (56%) had a qSOFA of 0 in 2020. Only 40 patients (4%) in 2019 and 28 patients (3%) in 2020 had a qSOFA of 3 at hospital admission.

The majority of patients were hospitalized during weekdays, with 734 patients (72%) admitted in 2019 and 700 patients (69%) admitted in 2020. The average length of stay (standard error) was 12 days (0.5 days) in 2019 and 10 days (0.3 days) in 2020. In 2020, 389 (36%) of the patients were initially admitted to the buffer ward, which was a ward where patients stayed while waiting for the result of the SARS-CoV-2 screening test. When the screening test was negative, the patients went to a regular internal medicine ward (Table 2).

**Table 2:** Characteristics of the hospitalized patients in the internal medicine ward of Coimbra Hospital and University Center between March and May of 2019 and 2020 CPD: Chronic pulmonary disease; qSOFA: Quick sequential organ failure assessment; RAS: Renin-angiotensin system; SE: Standard error. Health comorbidities specified correspond to comorbidities and not the admission/discharge diagnosis. The medications specified correspond to home medication and not the treatments given to patients during their hospital stay.

Variables	2019 (N = 1013; control group)	2020 (N = 1008; study group)	p-values
Age: Mean, in years (SE)	78 (0.43)	79 (0.45)	p = 0.1078
Gender: Female	508 (50%)	554 (55%)	p = 0.024
Katz index at hospital admission			
0	301 (30%)	286 (29%)	p = 0.622
1	76 (8%)	45 (4%)	p < 0.001
2	61 (6%)	26 (3%)	p = 0.001
3	51 (5%)	70 (7%)	p = 0.058
4	112 (11%)	221 (22%)	p < 0.001
5	75 (7%)	43 (4%)	p = 0.003
6	334 (33%)	310 (31%)	p = 0.335
qSOFA at hospital admission			
0	402 (40%)	558 (56%)	p < 0.001
1	372 (37%)	307 (30%)	p < 0.001
2	188 (19%)	111 (11%)	p < 0.001
3	40 (4%)	28 (3%)	p = 0.221
Mean	0.866	0.611	
Day of hospital admission: Week	734 (72%)	700 (69%)	p = 0.139
Time of hospital admission			
Between 8 am and 4 pm	231 (23%)	268 (27%)	p = 0.038
Between 4 pm and 0 am	497 (49%)	475 (47%)	p = 0.368
Between 0 am and 8 am	280 (28%)	265 (26%)	p = 0.311
Length of stay, in days (SE)	12 (0.5)	10 (0.3)	p < 0.001
Admission at buffer ward	-	389 (39%)	-
Death during hospitalization	122 (12%)	175 (17%)	p = 0.001
Health comorbidities			
Heart failure	371 (37%)	375 (37%)	p = 1.000
Diabetes mellitus	362 (36%)	331 (33%)	p = 0.156
CPD/asthma	176 (17%)	189 (19%)	p = 0.242
Hypertension	731 (72%)	706 (70%)	p = 0.322
Chronic kidney disease	207 (20%)	198 (20%)	p = 1.000
Dementia	227 (22%)	230 (23%)	p = 0.590
Cerebrovascular disease	208 (21%)	181 (18%)	p = 0.089
Cancer	190 (19%)	178 (18%)	p = 0.563
Medication			
Diuretics	534 (53%)	571 (57%)	p = 0.071
Beta-blockers	306 (30%)	371 (37%)	p < 0.001
Calcium channel blockers	183 (18%)	234 (23%)	p = 0.005
Statins	425 (42%)	422 (42%)	p = 1.000
Antiplatelets	213 (21%)	203 (20%)	p = 0.578
Anticoagulants	283 (28%)	299 (30%)	p = 0.322
Inhibitors of RAS	489 (48%)	503 (50%)	p = 0.369
Oral hypoglycemic medications	225 (22%)	215 (21%)	p = 0.584
Insulin	136 (13%)	129 (13%)	p = 1.000
Proton pump inhibitors	439 (43%)	413 (41%)	p = 0.362

The most commonly observed health comorbidities were hypertension (72% and 70% in 2019 and 2020, respectively), heart failure (37% in 2019 and 2020), and diabetes mellitus (36% and 33% in 2019 and 2020, respectively). The most frequent home medications were diuretics (53% and 57% in 2019 and 2020, respectively) and proton pump inhibitors (43% and 41% in 2019 and 2020, respectively) (Table 2).

In-hospital mortality among hospitalized patients

During hospitalization, 122 patients (12%) died in 2019 and 175 patients (17%) died in 2020. (Table 2). Among the deceased, the mean age (standard deviation) was 83 years (nine years) in 2019 and 83 (10 years) in 2020. Sixty-eight of the patients (56%) in 2019 were men, and 98 patients (56%) in 2020 were men. Concerning the patients’ functional status at hospital admission in this population, 86 (72%) had a Katz index under 3, and 14 (12%) were fully autonomous in 2019; 96 (55%) had a Katz index under 3, and 27 (15%) were fully autonomous in 2020. Regarding the severity of clinical presentation at hospital admission, the average qSOFA (standard deviation) was 1.4 (0.8) in 2019 and 1 (0.9) in 2020 (Table 3).

**Table 3:** Characteristics of the hospitalized patients that died in the internal medicine ward of Coimbra Hospital and University Center between March and May of 2019 and 2020 SD: Standard deviation; qSOFA: Quick sequential organ failure assessment.

Variables	2019 (N = 122)	2020 (N = 175)	p-value
Age: Mean, in years (SD)	83 (9)	83 (10)	p = 1.000
Gender: Female	54 (44%)	77 (44%)	p = 1.000
Katz index at hospital admission			
0	74 (62%)	74 (43%)	p = 0.001
1	9 (8%)	14 (8%)	p = 1.000
2	3 (2%)	8 (5%)	p = 0.182
3	4 (3%)	8 (5%)	p = 0.397
4	8 (7%)	36 (21%)	p = 0.001
5	7 (6%)	6 (3%)	p = 0.206
6	14 (12%)	27 (15%)	p = 0.460
qSOFA at hospital admission			
Mean (SD)	1.4 (0.8)	1 (0.9)	p < 0.001

Association between characteristics, health comorbidities, chronic medication, and in-hospital mortality

In univariate analysis, there was a significant association between in-hospital mortality and age in 2019 and 2020, in-hospital mortality and functional status in 2019 and 2020, and in-hospital mortality and severity at hospital admission in 2019 and 2020. Gender was found to have a significant association with in-hospital mortality only in 2020, with men having the highest mortality (OR = 1.71 [1.23, 2.37]). There was no statistically significant association between in-hospital mortality and the day of hospital admission or between in-hospital mortality and the time of hospital admission. In 2020, no statistically significant association was found between in-hospital mortality and prior admission to a buffer ward.

Concerning health comorbidities, there was a significant association between in-hospital mortality and the presence of dementia (OR = 2 [1.33, 3.01]) and cancer (OR = 1.57 [1.01, 2.44]) in 2019. In 2020, there was a significant association with dementia (OR = 2.32 [1.63, 3.29]), cerebrovascular disease (OR = 1.99 [1.36, 2.91]), and cancer (OR = 1.62 [1.09, 2.40]).

Considering the association between in-hospital mortality and home medication, there was a significant association with previous use of oral hypoglycemic medications (OR = 0.45 [0.26, 0.80]) in 2019 and previous use of inhibitors of the renin-angiotensin system (OR = 0.69 [0.50, 0.96]) in 2020 (Table 4).

**Table 4:** Univariate analysis for death according to the year of hospitalization CPD: Chronic pulmonary disease; RAS: Renin-angiotensin system; qSOFA: Quick sequential organ failure assessment.

Variables	2019 Odds ratio (95% CI)	p-values	2020 Odds ratio (95% CI)	p-values
Age	1.05 (1.03, 1.07)	p < 0.001	1.03 (1.02, 1.05)	p < 0.001
Gender	1.31 (0.89, 1.91)	p = 0.167	1.71 (1.23, 2.37)	p = 0.0013
Katz index at hospital admission	0.73 (0.66, 0.79)	p < 0.001	0.81 (0.76, 0.87)	p < 0.001
qSOFA at hospital admission	2.20 (1.77, 2.74)	p < 0.001	2.41 (1.72, 3.37)	p < 0.001
Day of hospital admission	0.84 (0.54, 1.30)	p = 0.437	1.39 (0.99, 1.96)	p = 0.058
Time of hospital admission	1.22 (0.78, 1.34)	p = 0,873	1.05 (0.84, 1.31)	p = 0.687
Admission to buffer ward	-	-	0.82 (0.59, 1.16)	p = 0.265
Heart failure	1.01 (0.68, 1.50)	p = 0.956	1.10 (0.83, 1.44)	p = 0.517
Diabetes mellitus	0.66 (0.44, 1.01)	p = 0.054	0.81 (0.57, 1.16)	p = 0.249
CPD/asthma	1.34 (0.84, 2.14)	p = 0.222	1.25 (0.84, 1.87)	p = 0.273
Hypertension	0.67 (0.45, 1.00)	p = 0.053	0.81 (0.57, 1.15)	p = 0.234
Chronic kidney disease	1.32 (0.84, 2.05)	p = 0.226	1.06 (0.77, 1.45)	p = 0.733
Dementia	2.00 (1.33, 3.01)	p = 0.001	2.32 (1.63, 3.29)	p < 0.001
Cerebrovascular disease	1.52 (0.98, 2.34)	p = 0.059	1.99 (1.36, 2.91)	p < 0.001
Cancer	1.57 (1.01, 2.44)	p = 0.046	1.62 (1.09, 2.40)	p = 0.016
Diuretics	1.34 (0.91, 1.96)	p = 0.138	1.22 (0.87, 1.70)	p = 0.250
Beta-blockers	1.10 (0.73, 1.65)	p = 0.652	0.87 (0.62, 1.23)	p = 0.434
Calcium channel blockers	0.65 (0.38, 1.14)	p = 0.132	0.67 (0.44, 1.01)	p = 0.057
Statins	0.79 (0.53, 1.16)	p = 0.227	0.84 (0.61, 1.15)	p = 0.276
Antiplatelets	1.12 (0.80, 1.57)	p = 0.524	1.12 (0.75, 1.67)	p = 0.568
Anticoagulants	0.97 (0.59, 1.39)	p = 0.654	1.11 (0.78, 1.57)	p = 0.574
Inhibitors of RAS	0.80 (0.55, 1.17)	p = 0.256	0.69 (0.50, 0.96)	p = 0.026
Oral hypoglycemic medications	0.45 (0.26, 0.80)	p = 0.006	0.69 (0.45, 1.06)	p = 0.091
Insulin	0.89 (0.50, 1.58)	p = 0.696	0.59 (0.34, 1.04)	p = 0.068
Proton pump inhibitors	1.21 (0.83, 1.77)	p = 0.318	1.37 (0.99, 1.90)	p = 0.058

Differences between in-hospital mortality and severity at hospital admission in 2019 and 2020

There was a statistically significant increase in in-hospital mortality in 2020 compared to 2019 (17.36% vs 12.04%, p-value < 0.001). The mean qSOFA at hospital admission was 0.611 in 2020 and 0.866 in 2019 (Table 2). There was a statistically significant difference between the severity of hospital admission in 2020 and 2019 (p-value < 0.001).

Predictors of in-hospital mortality

Table 5 shows the multivariable logistic regression for the outcome of death in patients without SARS-CoV-2 infection who were admitted to an internal medicine ward of a tertiary care hospital in Portugal between March and May 2019 and 2020.

**Table 5:** Multivariable logistic regression analysis for the outcome of “death” qSOFA: Quick sequential organ failure assessment; RAS: Renin-angiotensin system.

Variables	2019 Odds ratio (OR) (95% CI)	p-values	2020 Odds ratio (OR) (95% CI)	p-values
Age	1.02 (1.00, 1.05)	p = 0.022	1.03 (1.01, 1.05)	p = 0.01
Gender	-	-	2.17 (1.51, 3.12)	p < 0.001
Katz index at hospital admission	0.79 (0.71, 0.87)	p < 0.001	0.91 (0.84, 0.99)	p = 0.04
qSOFA at hospital admission	1.84 (1.43, 2.36)	p < 0.001	1.51 (1.23, 1.86)	p < 0.001
Chronic kidney disease	-	-	-	-
Dementia	0.98 (0.62, 1.55)	p = 0.921	1.63 (1.08, 2.46)	p = 0.02
Cerebrovascular disease	-	-	1.65 (1.10, 2.49)	p = 0.016
Cancer	1.99 (1.22, 3.23)	p = 0.006	1.70 (1.11, 2.62)	p = 0.015
Calcium channel blockers	-	-	-	-
Statins	-	-	-	-
Inhibitors of RAS	-	-	0.69 (0.49, 0.99)	p = 0.048
Oral hypoglycemic medications	0.45 (0.25, 0.81)	p = 0.007	-	-

According to the full model for 2019, the risk of death also increased with age. The odds of death were 0.79 times lower (95% CI: 0.71, 0.87) for each unit increase in the Katz index at hospital admission and 1.84 times higher (95% CI: 1.43, 2.36) for each unit increase in the qSOFA at hospital admission. In terms of health comorbidities and home medication, the odds of death were 1.99 times higher in the cancer group (95% CI: 1.22, 3.23) and 0.45 times lower in the group using oral hypoglycemic medications (95% CI: 0.25, 0.81).

Considering the full model for 2020, the risk of death increased with age. The odds of death were 2.17 times higher in the male group (95% CI: 1.51, 3.12). The odds of death were 0.91 times lower (95% CI: 0.84, 0.99) for each unit increase in the Katz index at hospital admission and 1.51 times higher (95% CI: 1.23, 1.86) for each unit increase in the qSOFA at hospital admission. Regarding health comorbidities and home medication, the odds of death were 1.63 times higher in the group with dementia (95% CI: 1.08, 2.46), 1.65 times higher in the group with cerebrovascular disease (95% CI: 1.10, 2.49), 1.70 times higher in the group with cancer (95% CI: 1.11, 2.62), and 0.69 times lower in the group using inhibitors of the renin-angiotensin system (95% CI: 0.49, 0.99).

**Figure 2:**
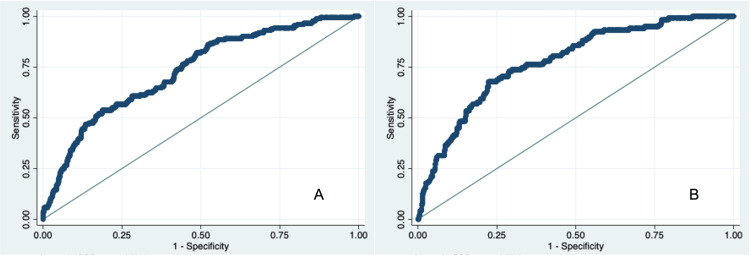
The area under the curve illustrating the performance of the full model to predict in-hospital mortality of patients without SARS-CoV-2 infection A: The area under the curve illustrating the performance of the full model to predict mortality of patients without SARS-CoV-2 infection admitted to an internal medicine ward of Coimbra Hospital and University Center between March and May 2020. AUC = 0.7311. B: The area under the curve illustrating the performance of the full model to predict mortality of patients without SARS-CoV-2 infection admitted to an internal medicine ward of Coimbra Hospital and University Center between March and May 2019. AUC = 0.7763. AUC: Area under the ROC curve; ROC: Receiver operating characteristic.

Figure 2 depicts the full model performance in predicting in-hospital mortality of patients without SARS-CoV-2 infection admitted to an internal medicine ward of a tertiary care hospital in Portugal between March and May 2019 and 2020. For 2019, the full model included age, Katz index, and qSOFA at hospital admission, cancer presence, and the use of oral hypoglycemic medications. For 2020, the full model included age, gender, Katz index, and qSOFA at hospital admission, dementia, cerebrovascular disease, cancer, and the use of renin-angiotensin system inhibitors. In the two years, a valuable discrimination capacity was observed, with an area under the ROC curve (AUC) of 0.7763 in 2019 and 0.7311 in 2020.

## Discussion

In Portugal, in-hospital mortality in the internal medicine wards is estimated to be between 8% and 15% [12]. Our work showed a significant increase in in-hospital mortality in 2020 (17.36%), suggesting that the COVID-19 pandemic held a meaningful impact on the treatment of patients without SARS-CoV-2 infection. This increase in in-hospital mortality in 2020 was in accordance with the international study conducted in Italy, which reported a relevant increase in all-cause mortality and also with the excess mortality projected in a study based on preliminary data from Portugal [2,13]. Nevertheless, the results of Nogueira et al. should be interpreted with caution because this excess mortality was attributed mainly to a decrease in access to healthcare, which may not solely explain the results observed, as pointed by other authors [14-17]. As far as we know, this is the first study to investigate the impact of the COVID-19 pandemic on in-hospital mortality in patients without SARS-CoV-2 infection in an internal medicine ward of a tertiary care hospital in Portugal during the first outbreak of the pandemic, resorting to the reality of one of the few medical specialties that have never seen its activity interrupted.

We observed a statistically significant difference in the severity at hospital admission with patients admitted in 2020 having a lower qSOFA compared to the patients admitted in 2019. This may be in line with the hypothesis raised by Melo et al., which indicated that the health system was failing toward the urgent needs of patients without SARS-CoV-2 infection, namely cancer patients under treatment or surveillance, as the number of deaths from all causes had increased when compared to previous years [11].

Our study population was identical to those described in previous works [18-20]. The average age was above 78 years, with a high prevalence of health comorbidities such as hypertension, diabetes, and heart failure exceeding 25%. As a result, the high prevalence of prescribed diuretics (more than 50% of our study population), renin-angiotensin system inhibitors (more than 40% of our study population), proton pump inhibitors (more than 40% of our study population), and statins (more than 30% of our study population) is remarkable.

In a regular internal medicine ward, age is an important predictor of in-hospital mortality, with reports showing that patients older than 90 years have mortality twice as high as those between 65 and 90 years [21]. The same result is seen when assessing the functional status at hospital admission [21]. Furthermore, several studies have reviewed the impact of health comorbidities such as heart failure and active malignancy on adverse outcomes in hospitalized patients, namely the length of stay and mortality, showing that the scores of comorbidities are important predictors of in-hospital mortality [18,21,22]. The impact of home medication on in-hospital mortality has also been studied, with studies identifying an association between the previous use of statins and antiplatelet drugs and lower in-hospital mortality rates in patients admitted due to sepsis [23,24]. Our findings suggest that previous use of renin-angiotensin system inhibitors and oral hypoglycemic medications was associated with a lower risk of in-hospital mortality, which should be interpreted with caution because it may represent a healthier user effect.

Furthermore, only a few reports have studied the impact of the day and time of hospital admission on in-hospital mortality. In fact, the identification of organizational factors that may influence the patients' outcomes during hospitalization at an institutional level is crucial for the control of the quality of healthcare. It is not expected that neither time nor the day of hospital admission is associated with in-hospital mortality if healthcare resources are maintained adequately. There are not many papers that report whether hospital admissions on weekdays or weekends have an impact on the mortality of hospitalized patients. Few published articles show conflicting findings [25-27]. Furthermore, few studies performed in the intensive care setting suggest that hospitalization after hours is not associated with increased mortality [27-29]. However, the trend in the internal medicine wards differs greatly from the intensive care unit setting. When comparing the two years studied, our study did not find a statistically significant difference in admission on weekdays or weekends. To the best of our knowledge, this is one of the first studies to address these variables in the setting of an internal medicine ward in a tertiary care hospital in Portugal. Nevertheless, studies aimed specifically at this critical question must be conducted.

Our study has important limitations. They include the retrospective design of the study and the possibility of the existence of a false negative polymerase chain reaction, with consequences in the underdiagnosis of deaths related to SARS-CoV-2 infection. The use of the qSOFA, which was developed in the context of sepsis, for assessing the severity of hospital admission is another limitation. Furthermore, it is important to be aware that our work addresses the reality of an internal medicine ward, whose patients have particular characteristics and health comorbidities, preventing the generalization of the conclusions presented to the reality of all hospital services. Despite these limitations, our study has several strengths, namely the sample size and the large number of variables that were taken into account for studying in-hospital mortality.

## Conclusions

In conclusion, our work showed a statistically significant increase in in-hospital mortality during the COVID-19 pandemic for patients without SARS-CoV-2 infection compared to the mortality rate in 2019 (our control group). This was not apparently explained by the differences in the characteristics of hospitalized patients, namely age, gender, health comorbidities, and home medication. The higher mortality of patients without SARS-CoV-2 infection in an internal medicine ward of a tertiary care hospital in Portugal during the pandemic compared to the year prior to the pandemic requires further investigation to identify the potential causes for this finding. Therefore, we hope that this study will be useful in the provision of rigorous support for future health policies regarding further waves of the COVID-19 pandemic or other humanitarian emergencies.
